# DNA‐induced unfolding of the thyroid hormone receptor α A/B domain through allostery

**DOI:** 10.1002/2211-5463.12229

**Published:** 2017-05-15

**Authors:** Elias J. Fernandez, Vandna Gahlot, Celeste Rodriguez, Jacob Amburn

**Affiliations:** ^1^ Biochemistry & Cellular and Molecular BiologyUniversity of TennesseeKnoxvilleTNUSA

**Keywords:** A/B domain, interdomain allostery, intrinsically disordered protein domain, nuclear receptor, protein–DNA interactions, thyroid receptor

## Abstract

The A/B domains of nuclear receptors such as thyroid receptor α (TRα) are considered to be conformationally flexible and can potentially adopt multiple structural conformations. We used intrinsic tryptophan fluorescence quenching and circular dichroism spectroscopy to characterize the unfolding of this A/B domain upon DNA binding to the contiguous DNA‐binding domain (DBD). We propose that this allosteric change in A/B domain conformation can allow it to make the multiple interactions with distinct molecular factors of the transcriptional preinitiation complex. We further suggest that by influencing the affinity of the DBD for DNA, A/B domain can fine‐tune the recognition of promotor DNA by TRα.

**Abbreviations**
CD: circular dichroism
DBD: DNA‐binding domain
DR4: direct repeat 4
ITC: isothermal titration calorimetry
LBD: ligand‐binding domain
NR: nuclear receptor
T3: triiodothyronine
TRE: thyroid receptor response element
TR: thyroid hormone receptor

The effects of the thyroid hormone (triiodothyronine, T3) are widespread in development, homeostasis and metabolism. The T3 receptors (thyroid hormone receptor, TR) are encoded by two closely related genes (α and β) 1. The T3Rα genes in humans express the T3‐binding isoform TRα1 2. The TRβ gene expresses TRβ1 and TRβ2, which differ only in their N‐terminal A/B regions, and are also distinct from the A/B region of TRα1 3. TRα is mostly expressed in the brain 4 and is associated with the development of the nervous system 5. TRα is constitutively localized within the nucleus where it interacts with nucleosomal DNA 6, 7. In the absence of T3 ligand, TRα is observed to actively repress transcription through interactions with transcriptional corepressors such as SMRT and NCoR 8, 9, 10.

Thyroid hormone receptors are members of the nuclear receptor (NR) superfamily of ligand‐mediated transcription factors 2. NRs have common modular structural features that include an N‐terminal domain (A/B domain, Fig. 1A). This A/B domain is of variable length and amino acid sequence and encompasses a ligand‐independent transactivation function (AF1) domain that is critical for regulating transactivation 11, 12. Following the A/B domain is a highly conserved DNA‐binding domain (DBD; C domain, Fig. 1A) that binds palindromic DNA sequences called hormone response elements (HRE). A short ‘hinge’ sequence (D domain) connects the DBD (C domain) to a C‐terminal ligand‐binding domain (LBD; E/F domain, Fig. 1A). Upon binding agonist‐ligands, the LBD (E/F domain) undergoes conformational changes which results in the recruitment of coactivator molecules 13, 14, 15, 16, 17. Antagonists and inverse agonists disrupt the ‘active‐state’ LBD and the resulting LBD conformation functions as a docking site for corepressors 18, 19, 20. Also, except for the A/B domains, the amino acid sequences of TRα and TRβ are over 90% identical. Since TRs differ most significantly in the N‐terminal A/B domain, it is suggested that this region plays a significant role in mediating the distinct roles of these receptors 21. It has also been proposed that TRα‐mediated transcriptional regulation can also occur through specific interactions of the A/B domain with the PIC, specifically with transcription factor IIB (TFIIB) 21, 22, 23, 24 and the TATA‐binding protein (TBP) 25. Transcriptional repression and similar interactions have also been observed between TRβ and TFIIB 21, 23, 26.

**Figure 1:**
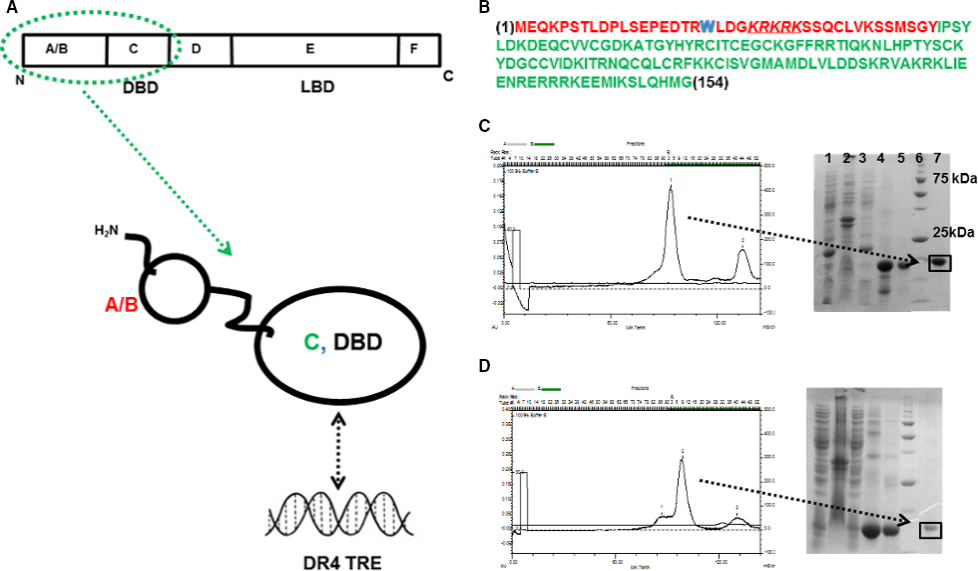
(A) NR domain topology displaying single‐letter domain assignments. The region circled in green (above) is the focus of this study and the structural topology shows the relative orientation of the domains with DR4 TRE DNA (below). (B) The amino acid sequence of the TRα A/B + C domain molecular construct is colour coded (A/B domain in red and C domain in green). The single tryptophan is shown in blue. (C and D) Results from the *Escherichia coli* overexpression and purification of the TRα (A/B + DBD) and TRα (DBD) molecular constructs, respectively. The molecular weight standards are on lane 6 (the positions of the 75 kDa and 25 kDa standards are labelled) and the purified proteins are in lane 7.

By and large, the N‐terminal domain of NRs is the least understood. This A/B region is diverse in size, sequence and is conformationally malleable 12, 27, 28, implying that this domain plays disparate roles in conferring cell type and/or promoter specificity 21. Moreover, there are no data on the atomic resolution structure of any NR A/B domain conformation to date.

Nuclear receptor structure is strongly affected by the presence and even sequence of the DNA response element 29. The source of these may result from conformational changes within the DBD as observed in structures of glucocorticoid (GR) bound to multiple GREs 30, 31. This may explain, in part, the DNA‐dependent interactions between the TRα DBD and LBD (E/F domain) reported earlier 32. DNA binding is also central to allosteric communication between the A/B and C (DBD) domains 27, 33, 34, 35, 36. Multiple DNA‐binding site sequences have been identified for TRα. TR isoforms and oligomers exhibit preferential binding to specific DNA sequences called thyroid response elements (TRE) 37. These TRE sequences consist of consensus AGGTCA (half‐sites) arranged as direct repeats (DR), palindromic sequences (Pal) or inverted palindromic sequences (IP), each with differing spacing between the half‐sites.

Allostery is a recognized regulatory feature within NRs such that ligand binding and even minor perturbations (such as nonbinding‐site mutations) are detected at distal regions of NRs 15, 16, 17, 27, 56, 57, 58, 59. With distinct structural changes, allostery has been observed to link ligand, coactivator and the DNA‐binding sites 17, 32, 42. Furthermore, DNA binding is also central to allosteric communications between the A/B and C (DBD) domains 27, 33, 34, 35, 36, 43. Increasingly, cooperative interactions between multiple NR domains have also been reported to modulate transactivation suggesting additional layers of regulation 32, 44.

Here, we report a notable conformational change in the TRα A/B domain that is initiated through allostery through the TRα DBD by DNA. The shorter, 50‐amino acid A/B domain of TRα encompasses several of the structural motifs that have been identified in NRs with significantly larger A/B domains to be important for ligand‐independent activity 24. Of these, distinct variations of the KRKRK amino acid sequence motif are common to several NRs including TR, progesterone (PR) and the liver X receptor (LXR) 28. We are able to observe that the TRα A/B domain can allosterically enhance the binding affinity of the receptor for direct repeat 4 (DR4) TRE DNA. Furthermore, using a combination of circular dichroism (CD) and intrinsic tryptophan fluorescence spectroscopy, we can report that the binding of DNA to the TRα DBD (C domain) induces unfolding within the flanking TRα A/B domain. Overall, these observations suggest a structural basis for intramolecular cooperativity within TRα that fine‐tunes binding to specific DNA sites.

## Experimental procedures

### Protein expression and purification

The chicken thyroid hormone receptor α1 gene (cTRα1, NCBI accession #: NP_990644.1) is over 90% identical to human TRα1 (NCBI accession #: NP_955366.1) at the amino acid level and is used for all experiments here. TRα (A/B + DBD, amino acid 1–154), TRα (DBD, amino acids 37–154) 32 and TRα (A/B domain, amino acids 1–50) were cloned into the plasmid pET15b (Life Technologies Inc., Carlsbad, CA, USA) to produce pET15b‐TRα (A/B + DBD), pET15b‐TRα(DBD) and pET15b‐TRα (A/B domain), respectively. Proteins were produced in *Escherichia coli* BL21 (DE3) RIPL cells. Protein synthesis was induced with 0.5 mm isopropyl β‐D‐thiogalactoside (IPTG) at 20ᵒC. Cells were lysed by sonication in 50 mm Tris, pH 8.0, 500 mm NaCl, 20 mm Imidazole, 10% glycerol, 1 protease inhibitor tablet, 5.7 mm β‐mercaptoethanol, 0.5 μm PMSF, 10 μm ZnCl_2_, 10 mm MgCl_2_, recombinant DNase 1 (10 U). 6XHis‐tagged TR (A/B + DBD) and TR (DBD) were purified using Ni‐NTA agarose (Qiagen®, Germantown, MD, USA) with 0.3 m Imidazole, 50 mm Tris, pH 8.0, 500 mm NaCl and 10% glycerol. Proteins were further purified by size‐exclusion chromatography (SEC) using S200 Superdex 16/60 column (GE Healthcare Life Sciences, Pittsburgh, PA, USA) in buffer consisting 50 mm 4‐(2‐hydroxyethyl)‐1‐piperazineethanesulfonic acid (HEPES), pH = 7.5 (at 25 ᵒC), 125 mm NaCl, 5 mm MgCl_2_, 1 mm tris (2‐carboxyethyl)phosphine hydrochloride; TCEP). Protein was analysed using SDS/PAGE. Protein concentration was determined using the Bradford Assay (BioRad^®^, Hercules, CA, USA).

### Preparation of DR4 TRE DNA adduct

19‐mer DNA oligos containing the thyroid hormone response element (TRE) consensus site (DR4: 5′‐CCAGGTCATTTCAGGTCAG‐3′, where the underlined sequence is the NR binding site) were commercially obtained (Life Technologies Inc.) as single‐stranded oligomers 45. Double‐stranded DR4 TRE was prepared by mixing the complementary strands in equimolar ratios to a final concentration of 2 mm, followed by heat denaturation at 95 ᵒC for 5 min and annealing by gradual cooling to room temperature.

### Isothermal titration calorimetry (ITC)

Thyroid receptor α (A/B + DBD) and TRα (DBD), purified by SEC, were used for isothermal titration calorimetry (ITC) measurements using VP‐ITC MicroCal™ (MicroCal Inc., Northampton, MA, USA). Protein and ligand were prepared in 50 mm HEPES, pH 7.5, 125 mm NaCl, 5 mm MgCl_2_ and 1 mm TCEP. For titration experiments, protein concentration ranged from 30 to 45 μm and ligand DR4 TRE: 5′‐CCAGGTCATTTCAGGTCAG‐3′ concentration ranged from 300 to 400 μm. Both protein and ligand were degassed for 5–10 min. The experiments were initiated by injecting 28 × 10 μL aliquots of DR4 TRE from the syringe into the calorimetric cell containing 1.5 mL of protein solution. All the titrations were performed at 25 °C and the buffer (pH adjusted to 7.5 at 25 °C). The change in thermal power as a function of each injection was automatically recorded using microcal origin software and the raw data were further processed to yield binding isotherms of heat released per injection as a function of molar ratio of DR4 TRE to TRα (A/B + DBD) or TRα (C domain). The data were acquired and processed using the microcal origin (MicroCal Inc.) software. Data were collected in triplicate.

### Fluorescence spectroscopy

Fluorescence emission spectra of purified TRα (A/B + DBD) in 50 mm HEPES, pH 7.5, 125 mm NaCl, 5 mm MgCl_2_, 1 mm TCEP were recorded at various concentrations of DR4 TRE. A total of 2 mL protein (2 μm) was used to which 2 μL of DR4 TRE (0–9.4 μm) was added for each scan. To monitor the effect of sample dilution due to DR4 TRE titrations into protein, equal volumes of buffer were titrated into 2 mL protein (2 μm). The spectra were monitored using a PerkinElmer‐LS 55 Fluorescence Spectrometer at excitation wavelength of 295 nm at 300 nm·min^−1^. Emission wavelength range was set at 310 nm to 450 nm, with slit width of 5.0 nm; 1 cm path length rectangular cuvettes were used to take all measurements at room temperature. The final fluorescence intensity change curve was a result of three averaged curves from individual experiments. The contribution of DR4‐TRE to the TRα (A/B + DBD) + DR4 TRE spectrum was corrected by subtracting the spectrum of TRα (A/B + DBD) + buffer. Since multiple studies have shown that two molecules of TR bind a single TRE DNA 32, 46, titration data curves were fitted to a two‐site binding, nonlinear regression fitting model by prism7 (GraphPad software, La Jolla, CA, USA, www.graphpad.com), where change in fluorescence intensity was plotted against increasing concentrations of DR4 TRE ranging from 0.0 μm to 9.4 μm (Fig. 1B.)

### Circular dichroism (CD)

Circular dichroism spectra of TRα (A/B + DBD) and TRα (DBD; in 50 mm sodium phosphate buffer, pH = 7.5–8.0, 80 mm NaCl, and 5 mm MgCl_2_, 1 mm TCEP) in the presence and absence of DR4 TRE DNA were recorded using a JASCO J‐815 CD spectrometer. Protein to DNA ratio was 1: 1.1 for all experiments. All spectra were collected at 100 nm·min^−1^ scan rate in 2 mm cuvettes maintained at 4 °C. The band width was 4 nm with data pitch 1 nm. CD spectra of buffer and DR4 TRE (4–5 μm) were also recorded separately as controls. Each spectrum shown is the result of 30 spectra accumulations, averaged and smoothed. All the spectra were corrected for the contributions of the buffer and TRE DR4 47. Mean residue ellipticity ([θ], (deg cm^2^ dmol^−1^) was calculated using the capito software 48.

## Results

Here, we present data from studies on a 154‐amino acid, two‐domain molecular construct that encompasses the contiguous A/B (N terminus) and the C domains (DBD) of TRα (Fig. 1A). The TRα A/B domain comprises approximately 50 amino acids with an evolutionary conserved KRKRK motif (Fig. 1B) consisting of multiple charged residues 21, 24. Additionally, this construct contains a single tryptophan residue that is conveniently located within the A/B domain (^19^ *Trp*) and adjacent to the KRKRK motif which has enabled us to monitor the local changes in conformation with steady‐state intrinsic tryptophan fluorescence spectroscopy. In summary, we present data on the structural conformation of the TRα A/B domain, the conformational changes in this domain that are transmitted by allostery when the DBD (C domain) binds DNA, and the effect of the A/B domain on DNA recognition and binding.

The structural topology of TRα is shown in Fig. 1A. The two TRα constructs – TRα (A/B + DBD) and TRα (DBD), were purified to homogeneity as monomers of TR (A/B + DBD; 20.1 kDa) and TR (DBD; 15.9 kDa; Fig. 1C,D).

### The TRα A/B ↔ DBD allostery influences the binding affinity for DNA

**Figure 2:**
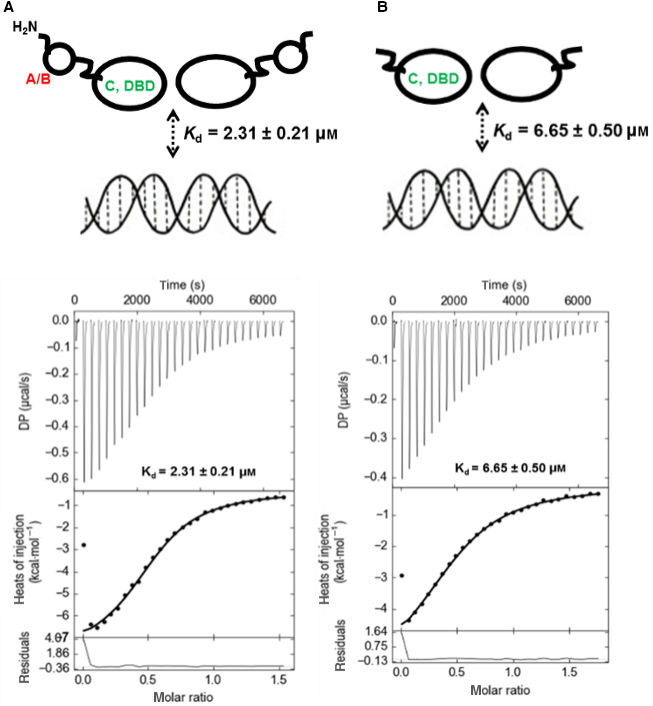
ITC measurements were performed to measure heat changes upon titrating DR4 TRE DNA into (A). TRα (A/B + DBD) and (B). TRα (DBD). For all titrations, the *c* values (*c* = *nK*_a_ *M*_tot_, where *n* is the stoichiometry parameter, *K*_a_ is the association constant = 1/*K*_d_ and *M*_tot_ is the concentration of the macromolecule, TRα) range from 6.5 to 9, which is within the ideal range for determining binding constants by ITC 73. Data obtained are summarized in Table 1.

The selectivity for DNA is central to the transcriptional activity of NRs. Here, we provide evidence that allostery between the TRα A/B domain and the DBD also occurs in reverse, i.e. TRα A/B domain can influence the behaviour of the TRα (C domain only) vis‐à‐vis its DNA‐binding affinity. Using ITC, we compare the binding affinity (*K*_d_) of TRα (A/B + DBD) domains and TRα (DBD) for DR4 TRE DNA. We observe a three‐fold increase in affinity of the intact TRα (A/B + DBD) domain for DR4 TRE DNA (*K*_d_ = 2.31 ± 0.21 μm) over the truncated TRα DBD (*K*_d_ = 6.65 ± 0.50 μm; Fig. 2). Also, the stoichiometry (*N*) of binding by both TRα (A/B + DBD) and TRα (DBD) is approximately *N* = 0.5 for TRE DR4, indicating that a single DR4 TRE binds two protein molecules. This is consistent with previous data showing two TR‐interacting half‐sites within the DR4 TRE 32, 45. Analyses of the thermodynamic parameters suggest that the TRα (A/B + DBD) ↔ DR4 TRE interaction is entropically less favourable (*T*Δ*S* = −2.05 kcal/mol) than the corresponding entropic contributions to the TRα (DBD) ↔ DR4 TRE interactions (*T*Δ*S* = 1.08 kcal·mol^−1^). Therefore, it is likely that the higher affinity between TRα (A/B + DBD) and DR4 TRE is directed by the approximately 1.6‐fold higher enthalpic contribution (∆*H* = −9.63 ± 1.20 kcal·mol^−1^) over the corresponding TRα (DBD) ↔ DR4 TRE interactions (∆*H* = −5.98 ± 0.43 kcal·mol^−1^; Table 1).

**Table 1:** Thermodynamic parameters of TRE DR4 interaction with TRα (A/B + DBD) and TRα DBD. Parameters are determined at 25 °C and pH = 7.5, as described in Experimental Procedures. The reported values are the average of three experiments and the errors are the standard deviation

Protein complexes	*K*_d_ (μm)	∆*H* (kcal·Mol^−1^)	*N* a	∆*G* (kcal·Mol^−1^)	*T*∆*S* (kcal·Mol^−1^)
TRα (A/B + DBD) + DR4	2.31 ± 0.21	−9.63 ± 1.20	0.54 ± 0.03	−7.68	−2.05
TRα (DBD) + DR4	6.65 ± 0.50	−5.98 ± 0.43	0.53 ± 0.02	−7.06	1.08

a The apparent stoichiometry from the curve fitting data.

### TRE binding to the DBD can influence specific local conformation of the A/B domain

**Figure 3:**
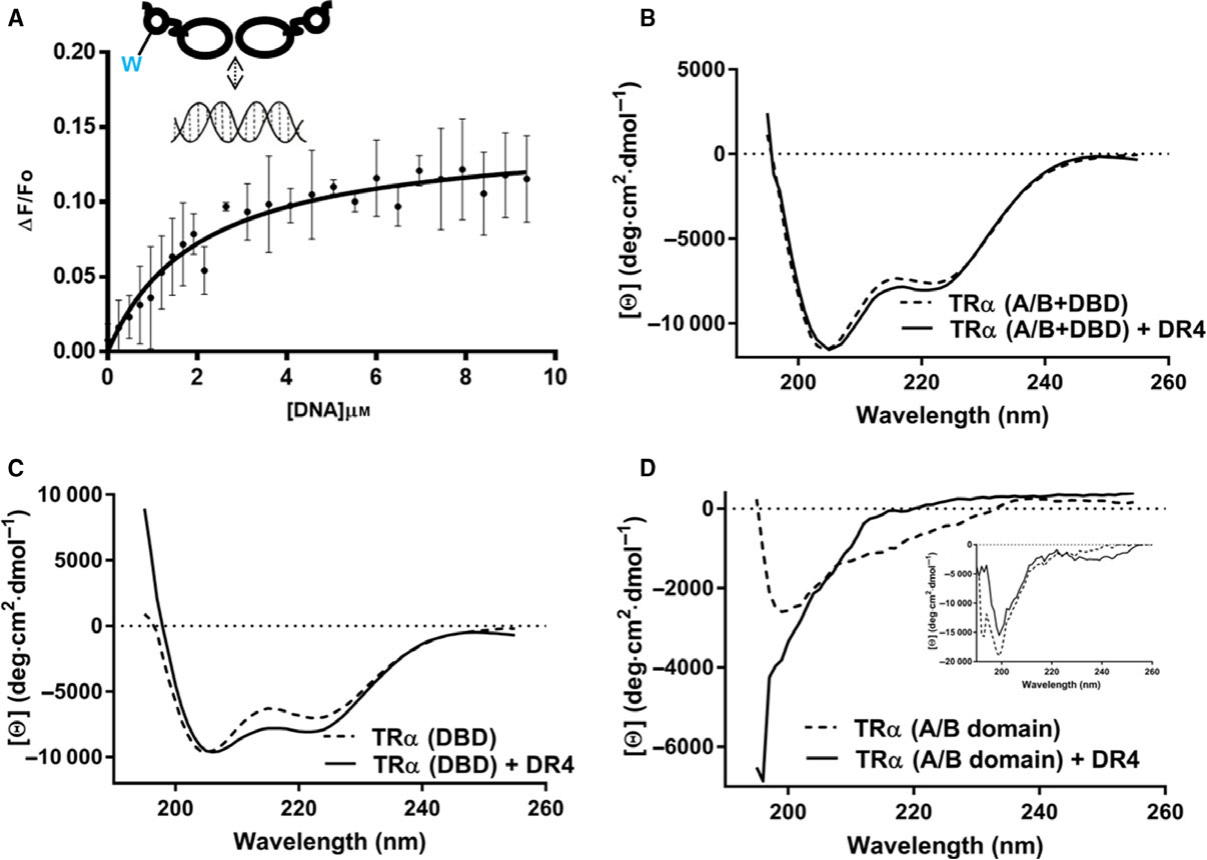
Conformational changes determined by Fluorescence and CD spectroscopy. (A) Change in intrinsic tryptophan fluorescence of TRα (A/B + DBD) is monitored in response to increasing levels of DR4 TRE DNA. The data above are obtained after subtracting buffer and DR4 TRE DNA contributions. In addition, no static quenching of molecular *Trp* was observed by DR4 TRE DNA. (B and C) Raw CD spectra of TRα (A/B + DBD) and TRα (DBD), respectively, ± DR4 TRE DNA. (D) The CD ([θ], (deg cm^2^ dmol^−1^) vs. wavelength, nm) spectra of the TRα (A/B domain) was calculated by individually subtracting the [θ] values for TRα (DBD) from TRα (A/B + DBD), for each corresponding wavelength, ± DR4 TRE DNA, respectively. The assumption made is that the conformations of the TRα (C domain), ± DR4 TRE DNA, are the same in both TRα (A/B + DBD) and TRα (DBD). Inset, CD spectra of TRα (A/B domain) measured directly ± DR4 TRE.

Our studies above indicate that there is an allosteric pathway that links the DNA‐binding site within the TRα DBD to the N‐terminal TRα A/B domain (Fig. 2). Here, we sought to determine if the DNA‐dependent allosteric communication between TRα A/B ↔ DBD is manifested in measurable conformational changes, specifically within the TRα A/B domain. Fortuitously, there exists only a single *Trp* residue within the entire TRα (A/B + DBD) molecular construct. Furthermore, at position 19 this ^19^ *Trp* is also both midway within the TRα A/B domain (residues 1–50) and distal from the DNA‐binding TRα DBD (residues 51–154; Fig. 1B). Thus, this single *Trp* enables us to directly identify conformational changes within the central region of TRα (A/B + DBD). *Trp* fluorescence quenching has been a common indicator of local and global conformational changes within the NR A/B domains 35, 49, 50 and due to allostery 16. We monitored the dose‐dependent changes in intrinsic steady‐state tryptophan fluorescence, accompanied by an approximately 5 nm red‐shift in fluorescence maxima, within TRα (A/B + DBD) in the presence of DR4 TRE (Fig. 3A). The measurable decrease in fluorescence suggests a specific change in the ^19^ *Trp* conformation, and furthermore, the conformational changes within the ^19^ *Trp* sidechain are more likely from a progressive decrease in its local hydrophobic environment, presumably from an increased exposure to the surrounding buffer 16. These titrations were also analysed to provide a quantitative measure of binding affinity: since the two DR4 half‐sites are indistinguishable for binding TRα 32, the average binding affinity of TRα (A/B + DBD) for DR4 TRE is *K*_d_ = 2.69 ± 0.22 μm. This binding constant confirms data obtained by calorimetry.

### TRE binding to the DBD results in unfolding of the TRα A/B domain

The spectroscopic analyses above suggest an allosteric conformational change within the TRα A/B domain upon binding DNA at the TRα(DBD). To determine the specific DNA‐dependent changes in structure within the TRα A/B domain, we utilized CD spectroscopy. Given that minor changes in the secondary structure of proteins can be detected in the raw CD spectra (θ in rad cm^−1^ vs. wavelength in nm) in the far‐UV (λ = 190–260 nm) range, we compared the CD spectra of the TRα (A/B + DBD) domains with TRα (DBD) in the absence and when complexed with DR4 TRE (Fig. 3B). For the TRα (DBD), there is a prominent change in the minima at 208 nm and 222 nm of the CD spectrum in the presence of DNA, which suggests a significant increase in α‐helical structure of the TR(DBD) upon binding DNA (Fig. 3C). Such conformational changes in NR DBDs have been previously observed using NMR spectroscopy confirming a dosage‐dependent stabilization of the NR DBD upon binding DNA 51, 52, 53, 54, 55. In this study, the CD spectra of TRα (A/B + DBD) indicates that while the TRα segment is predominantly α‐helical, the complexation of TRα (A/B + DBD) with DNA results in a markedly smaller change in secondary structure from the DNA‐free protein when compared with the corresponding structural changes within the TRα DBD‐only (Fig. 3C). To determine the source of this discrepancy between the TRα (A/B + DBD) and TRα DBD, we subtracted the spectroscopically measured molar ellipticity of CD of the TRα(DBD) from the TRα (A/B + DBD) domain. The resulting spectrum estimates the ‘calculated’ molar ellipticity ([θ], (deg cm^2^ dmol^−1^), and therefore the conformational change, of the TRα (A/B domain) within the TRα (A/B + DBD):DNA complex (Fig. 3D). Additionally, we do not detect significant secondary structure changes to the isolated TRα (A/B domain) in the presence of DR4 TRE (Fig. 3D inset). Taken together, these results suggest that the TRα (A/B domain) has partial α‐helical secondary structure within the ‘DNA‐free’ TRα (A/B + DBD). Upon binding DNA, the contiguous A/B domain and the DBD undergo contrasting conformational changes – while the A/B domain appears to convert from a more structured to a conformationally less‐rigid state, the DBD becomes conformationally more stable. Overall, this α‐helical‐to‐random coil unfolding of the TRα A/B domain appears to counteract the propensity for greater α‐helicity within the TRα(DBD) upon binding DR4 TRE. This may explain, in part, the smaller overall change in TRα (A/B + DBD) in comparison with the TRα(DBD), upon binding DR4 TRE.

## Discussion

Multiple lines of evidence suggest that the NR A/B domains are flexible and can adopt distinct conformations through allostery initiated by DNA:DBD interactions 12, 34, 43, 49, 50, 56, 57, 58, 59. A common observation is that the A/B domains in all NRs studied to date, the DNA‐initiated allostery elicits an increase in secondary structure (mostly α‐helicity) of this domain.

Multiple attempts to determine the structures of full‐length NRs have failed to identify the conformation of their N‐terminal domains 60. Yet, all these structures have indicated that there is no apparent direct interaction between the A/B domain and the DBD. Our observations suggest that DNA‐dependent conformational changes within the TRα A/B domain are distinct from the corresponding changes within the other NR A/B domains listed above. The implications for the unique mode of TRα A/B domain ↔ DBD allostery are broad. For instance, the TRα A/B domain is reported to interact with several cellular cofactors including TFIIB 21, 22, 23, 24 and TBP 25. Similar interactions have been observed between NRs and the PIC, such as the androgen (AR) 61, 62, COUP‐TF 63, oestrogen (ER) 63, 64, GR 65, mineralocorticoid (MR) 66 and PR 34, 63 receptors, among others. In each of these NRs, and distinct from TRα as reported here, the A/B domain is constrained to a more folded conformation by DNA‐allostery. This more‐structurally constrained A/B domain is observed to enhance the NR↔cofactor interaction.

In TRα, the sequence of basic residues ^23^KRKR^27^K has been identified to make specific interactions with TFIIB (Fig. 1B) 24). Adjacent to this basic motif is ^*19*^ *Trp*, which we show here by DR4 TRE DNA dose‐dependent fluorescence quenching to undergo conformational changes to a more exposed environment and this would be expected with the unfolding of this region of the TRα A/B domain. From truncation and associated binding studies, the corresponding TRα‐interacting domain of TFIIB is identified to be contained within residues 178–201 of an amphipathic α‐helix 24. Curiously, this TRα‐interacting TFIIB α‐helix has also separately been identified as integral to the binding interface between TFIIB and DNA 67. Together, these studies suggest that the formation of the TRα:TFIIB and the TFIIB:DNA complexes are mutually exclusive and that binding to TRα can disrupt the TFIIB‐DNA complex. In the absence of direct structural data, it is tempting to speculate that the DNA‐induced unfolding of the TRα A/B domain plays a role in inserting itself into the TFIIB‐DNA complex and the newly created TRα:TFIIB is stabilized by both interactions made by the charged ^23^KRKR^27^K and through the exposed apolar backbone of the TRα A/B domain. Indeed, such DNA‐induced unfolding events are less commonly reported in the literature and the Ets‐1 transcription factor is a singular prior example of an analogous DNA‐induced unfolding within a flanking domain through allostery 68, 69. In Ets‐1, this induced unfolding is proposed to ameliorate inhibitory intramolecular interactions and encourage intermolecular interactions that promote gene transcription.

Additionally, this study reinforces the observation that DNA recognition is finely tuned by the domains flanking the NR DBD. In both DNA‐bound TRα:RXR heterodimeric 45 and the TRβ monomeric 46 structures, the conformation of the TR DBD is virtually identical, suggesting a generic mechanism for DNA recognition and binding. Yet, using DBD and DBD‐LBD constructs of TRα, we have earlier established that the affinity of the DBD for DNA can be modulated through intramolecular allostery 32. Moreover, even subtle changes within these flanking domains (A/B or E/F domains) such as mutations 70 and interactions with cellular factors 32 or small‐molecule ligands 71 can affect DNA binding. Given the distinct unfolding process of the TRα A/B domain, the mechanism by which this domain can allosterically influence DBD↔DNA interactions is likely to be different from those of AR 35 and PR 72.

In summary, our data here suggest a distinct consequence of allostery within TRα. The data from CD spectroscopy show that conformational changes induced within the TR(DBD) are transmitted ‘upstream’ to the flanking A/B domain. The resultant conformation of the TRα A/B domain is less ordered within the intact, DNA‐bound TRα (A/B + DBD) than in the absence of DNA. This unfolding results in the repositioning of ^19^ *Trp* observed from the quenching of tryptophan fluorescence. The unusual feature of DNA‐induced, allosterically driven conformational changes within the TRα A/B domain is the overall loss in secondary structure, quantified as a decrease in its α‐helicity. Finally, this study showcases the diversity in the structural response to allostery within the NR superfamily. We are drawn to hypothesize that such structural responses have been evolutionarily selected to optimize the specific behaviour of individual members of these NR transcription factors.

## Author contributions

EJF designed and performed experiments, analysed data and wrote the manuscript. VG performed experiments and analysed data and CR and JA performed experiments.
